# Staying alert with polyhydramnios; an Ondine syndrome case

**DOI:** 10.1515/crpm-2022-0026

**Published:** 2023-04-27

**Authors:** Maria Pellisé-Tintoré, Anna Lucia Paltrinieri, Anna Abulí, Elena Murillo, Ariana Serrano, Gerard Albaigés

**Affiliations:** Department of Obstetrics, Gynaecology and Reproduction, Dexeus Universitary Hospital, Barcelona, Spain; Department of Neonatology and Pediatrics, Dexeus Universitary Hospital, Barcelona, Spain; Unit of Medical Genomics, Department of Obstetrics, Gynaecology and Reproduction, Dexeus Universitary Hospital, Barcelona, Spain

**Keywords:** amniotic fluid index, apneas, low fetal growth, Ondine’s curse, PHOX2B, polyhydramnios

## Abstract

**Objectives:**

Amniotic fluid is essential for proper fetal development. In the case of severe polyhydramnios associated with low fetal growth, a number of different underlying disorders must be considered. One such condition is congenital central hypoventilation syndrome (CCHS) or Ondine’s curse, a rare genetic disease caused by mutation of the PHOX2B gene. The incidence of CCHS is estimated to be 1 case in 200,000 live births. No publications have been made to date on the intrauterine period findings. This precludes an early intrauterine diagnosis and impedes ethically responsible therapeutic options.

**Case presentation:**

A 37-year-old patient presented in her second pregnancy with a small for gestation fetus and severe polyhydramnios evidenced in the third trimester ultrasound (US) study. There were no previous signs of maternal diabetes or fetal abnormalities at US. During the immediate postpartum period, the newborn presented repeated apneas with cyanosis and hypo-responsiveness. Neonatal arterial blood gas testing revealed severe respiratory acidosis requiring orotracheal intubation and admission to the Neonatal Intensive Care Unit. Over the following days, all imaging and functional test findings were within normal ranges. A *de novo* pathogenic PHOX2B variant was identified.

**Conclusions:**

Despite a high mortality rate, no neurological sequelae or other systemic diseases were recorded, thanks to multidisciplinary and coordinated follow-up.

## Introduction

Amniotic fluid (AF), with its multiple functions, is a nutrient reservoir with antibacterial properties that provides space, fluid and growth factors, all of which allow proper fetal development [1, 2]. In the absence of a correct balance between AF production and clearance, different conditions can arise, affecting adequate fetal development [3]. Polyhydramnios is defined as an abnormal increase in AF volume, of and usually appears in the second or third trimester of pregnancy [3, 4]. The prevalence of polyhydramnios varies from 1–2 %, depending on the method used for its diagnosis [5].

In clinical practice, polyhydramnios is evidenced by ultrasound (US) visualization of AF volume [4]. Two standardized quantitative measures for diagnosing and quantifying polyhydramnios are an Amniotic Fluid Index (AFI) greater than 24 cm or a single Deepest Vertical Pocket (DVP) greater than 8 cm [1, 3, 4]. More severe degrees of polyhydramnios are usually linked to a significantly increased risk of fetal abnormalities [3, 4, 6]. According to the Society for Maternal Fetal Medicine (SMFM), an AFI ≥35 cm is associated with a 20–40 % risk of fetal abnormality or genetic syndrome [3]. Therefore, when polyhydramnios appears, prompt assessment of the underlying etiology is required. In cases of severe polyhydramnios with associated fetal growth restriction, a genetic study should be carried out.

We present a case of severe polyhydramnios (AFI 41 cm) in an asymptomatic patient with normal study findings according to protocol. After birth, the newborn (NB) presented repeated apneas with cyanosis and hypo-responsiveness requiring orotracheal intubation (OTI) and admission to the Neonatal Intensive Care Unit (NICU). All morphological and functional tests proved normal.

## Case presentation

We present a 37-year-old woman with no known allergies and a history of full-term vaginal delivery four years ago, without complications. In the early third trimester ultrasound (US) control of her second pregnancy, increased AF was observed, with AFI of 41 cm (severe polyhydramnios).

**Figure 1:**
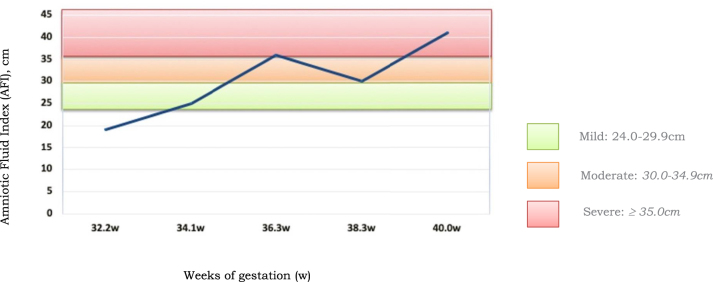
Amniotic fluid assessment during third trimester US.

Pregnancy was through an assisted reproduction technique due to asthenozoospermia, a primary male sterility factor. During the first trimester, combined screening for chromosomal abnormalities was performed, resulting in low risk. In the second trimester, the morphological US control showed no fetal abnormalities, and the patient presented no signs of maternal diabetes. However, during the third trimester, we started to observe an increase in AF, with an AFI of 25 cm (mild polyhydramnios) at 34+1 weeks of gestation (w). The AFI levels subsequently continued to increase, with AFI 36 cm at 36+3w and 41 cm at 40w (severe Polyhydramnios) (Figure 1).

We observed a fetal growth percentile below average, becoming small for gestational age (9th percentile) at 36+3w. None of the morphological US controls evidenced fetal abnormalities. Serological TORCH studies and Coombs testing proved negative. Amniocentesis with a chromosomal microarray analysis was offered, but the patient declined it, as the optimal timeframe for therapeutic options was limited.

The case was presented to the Maternal and Fetal Committee, which decided to induce labor at 40 weeks for better control and management. After eighteen hours of Prostaglandins and Oxytocin, cesarean section was indicated due to failed induction. A male infant was born in good condition, and with no need for any support. The Apgar score was 8–9 at 1 and 5 min, and the fetal pH 7.22 and 7.32. Birth weight was 3,700 g (75th centile). At 20 min of age, the newborn began to present repeated apneas with cyanosis, hypo-rresponsiveness and saturation that decreased from 100 to 70 % with recovery after stimulation, but with subsequent repetition of the apneas.

Neonatal arterial blood gas testing revealed severe respiratory acidosis requiring admission to the NICU, with non-invasive respiratory support in the form of nasal CPAP at first, followed by OTI at 3 h of age. Multiple studies were made over the following 24–48 h: basic bloods test, metabolic analyses, chest X-ray, pulmonary ultrasound, cerebral ultrasound, brain MRI, EEG, ocular fundus examination and all results being within normal ranges. Elective extubation attempts were made; however, reintubation proved necessary due to repeated episodes of apnea and acidosis.

Following a multidisciplinary discussion including Neonatology, Neurology and Genetics, a central hypoventilation syndrome was suspected. Finally, three months after birth, a *de novo* pathogenic *PHOX2B* variant was identified. The newborn is currently 2 years old requiring mechanical ventilation through positive pressure ventilation (PPV) via tracheostomy. To date, no neurological sequelae or other systemic disorder have been identified. Hope and future investigations are steer towards electrodes implanted near the phrenic nerve allowing freedom and mobility.

## Discussion

Congenital central hypoventilation syndrome (CCHS) also known as Ondine syndrome, is a rare genetic disease typically inherited in an autosomal dominant manner [7, 8]. *PHOX2B* is the only gene in which pathogenic variants are known to cause CCHS. This gene is expressed in the cells of the neural crest, affecting the autonomic and enteric nervous system [8]. Approximately 90 % of all patients with CCHS have a mutation in exon 3 of the *PHOX2B* gene that normally presents a repeat of 20 alanines. Such mutation is due to an increase in the number of these alanine repeats from 20 to a range of 24–33 alanines, and is known as poly-alanine repeat expansion mutations (PARMs) [9]. The rest of the patients have loss of function variants of the *PHOX2B* gene not related to PARMs. In our case we found 26 copies of CGN in one allele and 20 copies in the other one. A genotype-phenotype correlation emerged since a higher number of alanine repeats is associated with severe disease and the need for respiratory support [9].

CCHS is characterized by decreased autonomic control of spontaneous respiration, leading to hypoventilation during sleep. Treatment requires a multidisciplinary approach, with lifelong ventilation support being a key element. Congenital central hypoventilation syndrome is a multisystemic disorder with an increased risk of tumors of neural crest origin (in about 5–10 % of all patients) and is linked to other diseases such as Hirschsprung Disease in almost 50 % of all cases [8, 9]. Therefore, an early diagnosis is needed to ensure close monitoring of these patients. In our case, thanks to the prompt suspicion and rapid action of the Neonatology Department, the NB has not suffered neurological sequelae secondary to hypoxemia, and no other systemic disorder have been identified to date. The warning sign manifesting during pregnancy is isolated severe polyhydramnios. In effect, polyhydramnios must be considered a warning sign since it is an independent risk factor for fetal and perinatal mortality [10]. We could hypothesize that intrauterine apnea episodes during sleeping periods could be linked to lower swallowing of AF. Swallowing and breathing are both under the control of the brainstem. Decreased swallowing would consequently reduce AF elimination, while AF production would remain intact, since fetal urine and lung fluid are not affected. Consequently, the AF volume would increase, reaching severe levels [1, 2]. Jointly, these apneas episodes could also alter correct fetal development and growth.

A genetic study was offered to the parents which they finally declined. However, it is important to consider that even if an array or karyotype had been completed, no mutation would have been found. Next-generation sequencing (NGS) based technologies are unable to detect polyalanine repeat expansions. However, genetic studies can be of predictive value. Mangels et al. reported genetic disorders in almost 50 % of prenatal severe polyhydramnios cases [11].

Our exceptional case describes the intrauterine symptoms of a rare genetic disease with serious postnatal comorbidities. Faced with severe isolated polyhydramnios, we must investigate not only possible fetal malformations but also genetic or chromosomal disorders. Antenatal genetic testing must be considered in early isolated severe polyhydramnios with the purpose of anticipating guidance for prenatal and postnatal care. Hence, we would be able to forestall and establish an early prenatal diagnosis to present our patients with ethical and responsible options.
